# Withaferin A Synergizes the Therapeutic Effect of Doxorubicin through ROS-Mediated Autophagy in Ovarian Cancer

**DOI:** 10.1371/journal.pone.0042265

**Published:** 2012-07-30

**Authors:** Miranda Y. Fong, Shunying Jin, Madhavi Rane, Raj K. Singh, Ramesh Gupta, Sham S. Kakar

**Affiliations:** 1 Department of Physiology and Biophysics, University of Louisville, Louisville, Kentucky, United States of America; 2 Department of Medicine, University of Louisville, Louisville, Kentucky, United States of America; 3 Vivo Biosciences Inc., Birmingham, Alabama, United States of America; 4 Department of Pharmacology and Toxicology, University of Louisville, Louisville, Kentucky, United States of America; 5 James Graham Brown Cancer Center, University of Louisville, Louisville, Kentucky, United States of America; Harvard Medical School, United States of America

## Abstract

Application of doxorubicin (Dox) for the treatment of cancer is restricted due to its severe side effects. We used combination strategy by combining doxorubicin (Dox) with withaferin A (WFA) to minimize the ill effects of Dox. Treatment of various epithelial ovarian cancer cell lines (A2780, A2780/CP70 and CaOV3) with combination of WFA and Dox (WFA/DOX) showed a time- and dose-dependent synergistic effect on inhibition of cell proliferation and induction of cell death, thus reducing the dosage requirement of Dox. Combination treatment resulted in a significant enhancement of ROS production resulting in immense DNA damage, induction of autophagy analyzed by transmission electron microscope and increase in expression of autophagy marker LC3B, and culminated in cell death analyzed by cleaved caspase 3. We validated combination therapy on tumor growth using an in vitro 3Dimension (3D) tumor model and the more classic *in vivo* xenograft model of ovarian cancer. Both tumor models showed a 70 to 80% reduction in tumor growth compared to control or animals treated with WFA or Dox alone. Immunohistochemical analysis of the tumor tissues from animals treated with WFA/Dox combination showed a significant reduction in cell proliferation and formation of microvessels accompanied by increased in LC3B level, cleaved caspase 3, and DNA damage. Taken together, our data suggest that combining WFA with Dox decreases the dosage requirement of Dox, therefore, minimizing/eliminating the severe side effects associated with high doses of DOX, suggesting the application of this combination strategy for the treatment of ovarian and other cancers with no or minimum side effects.

## Introduction

Ovarian cancer is the most lethal malignancy of the female reproductive tract [1]. Due to lack of symptoms at an early stage of the disease, the five-year survival rate is only 27.2% [1]. The mainline treatment of ovarian cancer is cytoreductive surgery followed by platinum-based chemotherapy [2]. Initially, ovarian cancer responds positively in 70 to 80% of the cases [3]. However, within 18 to 24 months after initial treatment, tumor relapse occurs, which (for approximately 70% of patients) is attributed to the carcinomas having become platinum-resistant [3] This poor survival rate for women with platinum-resistant ovarian carcinomas points to an urgent need for an alternative treatment strategy.

Doxorubicin (Dox) is a broad-spectrum anthracylin isolated from *Streptomyces peucetius* that has been used for the treatment of several cancers, including ovarian, breast, and prostate [4]. In fact, anthracylins are the most widely used FDA approved anticancer drug [5]. Dox’s effectiveness has been attributed to its ability to intercalate between the DNA strands to act as a topoisomerase II inhibitor and/or bind covalently to proteins involved in DNA replication and transcription [5]. The use of Dox is limited by severe dose-dependent side effects including acute nausea and vomiting, stomatitis, neurological disturbances, myocardial toxicity, alopecia, and bone marrow aplasia [5]. Alternately, pegylated liposomal doxorubicin (PLD) (DOXIL) is regarded as one of the standard treatment options in recurrent ovarian cancers (ROC) [6]. Despite comparatively lower side effects, Doxil has very low response rate (<20%) [6].

More recently combination therapy with Dox has garnered more attention. Combining Dox with sildenafil resulted in an enhanced cell death through the down regulation of Bcl-2 coupled to increased caspase 3 through the enhancement of Dox-induced generation of reactive oxygen species (ROS) while attenuating Dox-induced cardiac dysfunction [7]. Dox has also been combined with HO-3867, a synthetic curcumin analog, to achieve enhanced cell death and reduced myocardial toxicity through the use of lower doses of Dox [8]. Therefore, combination therapy has proven to be a useful method to reduce the side effects associated with Dox while still retaining its therapeutic function.

Withaferin A (WFA) is bioactive, cell permeable steroidal lactone having withanolide skeleton as its basic structure. WFA is isolated from the plant *Withania somniferia*, which has been a part of Indian Ayurvedic medicine for centuries and is now available as an over-the-counter dietary supplement in the U.S. It has been used to treat a variety of conditions due to its anti-inflammatory and anti-bacterial properties. More recently, it has been suggested as a potential anti-cancer compound as it has been shown to inhibit tumor growth, angiogenesis, and metastasis [9], [10]. Several biological functions have been influenced by WFA including induction of apoptosis through inactivation of Akt and NF-κB [11] as well as decrease of pro-survival protein Bcl-2 [12], [13], induction of Par-4 [14], inhibition of Hsp90 and Notch-1 [15], G2/M cell cycle arrest [16], FOXO3a and Bim regulation [9], generation of ROS [17], [18] and down regulation of expression of HPV E6 and E7 oncoproteins [19].

A previous study has shown that WFA enhances the cytotoxic effect of Dox in an osteogenic sarcoma (U2OS) and breast cancer cell line (MCF-7) using a cell proliferation assay [20]. However, the combined effect of Dox and WFA has not been studied in ovarian cancer, a mechanism of action determined, or combination treatment tested *in vivo* for the suppression of tumor growth. We proposed that WFA when combined with Dox will elicit a synergistic effect on the suppression of ovarian tumor growth. To test our hypothesis, we studied the combined effect of Dox and WFA on cisplatin-sensitive ovarian epithelial cancer cell line A2780, cisplatin-resistant ovarian epithelial cell line A2780/CP70, and p53 mutant ovarian epithelial cell line CAOV3. For the first time we showed that cell death was induced by ROS production and DNA damage, leading to the induction of autophagy and culminating in cell death in caspase 3 dependent manner. We also showed that the effect of Dox and WFA *in vitro* using 3D tumors generated from A2780 cells on a human extracellular matrix. Furthermore, we examined the effect of combination treatment *in vivo* on tumor growth, proliferation, angiogenesis, autophagy, cell death, and DNA damage using xenograft tumors produced by injecting A2780 cells in nude mice.

## Materials and Methods

### Ethical Statement

Animals work reported in the manuscript was performed after approval of the protocol by University of Louisville Animal Care Use Committee (IACUC).

### Cell Culture

Human epithelial ovarian tumor cisplatin-sensitive (A2780) cell line was obtained as a gift from Dr. Denise Connolly (Fox Chase Cancer Center, Philadelphia, PA). The cell line was originally generated from human ovarian cancer patient prior to treatment [21]. The cisplatin-resistant (A2780/CP70) cell line was obtained as a gift from Dr. Christopher States (University of Louisville, Louisville, KY). This cell line was derived from A2780 cell line after treatment with cisplatin [22]. CAOV3 cell line was purchased from American Type Culture Collection (ATCC). A2780 and A2780/CP70 cells were cultured in RPMI medium containing 10% FBS, 1% Penicillin/Streptomycin, and 0.05% (v/v) Insulin (Sigma). CAOV3 cells were cultured in DMEM medium containing 10% FBS and 1% Penicillin/Streptomycin. Antibodies to phospho-BAD Ser^136^, Bcl-xL, cleaved caspase 3, and GAPDH were purchased from Cell Signaling Technology. Ki67 antibody was purchased from Santa Cruz Biotechnology, CD31 and LC3B from AbCam. Doxorubicin, withaferin A, N-acetyl-L-cysteine, superoxide dismutase, catalase, and DMSO were purchased from Sigma.

### Cell Proliferation (MTT) Assays

A2780, A2780/CP70, and CAOV3 cells growing in growth phase were trypsinized and were seeded into 96-well plates. After 24 h of plating, cells were treated in triplicates with various doses of Dox and WFA both alone or combination of WFA/Dox for 24, 48, and 72 h. After specified time, 20 µl MTT reagent from the cell proliferation assay kit (Promega) was added to each well and incubated for approximately 1 h. Color development was assessed by an ELISA reader at 492 nM as described previously [23]. Dox was solubilized in water and WFA was solubilized in DMSO. DMSO (0.1% v/v) was used as a vehicle control.

### Isobologram Analysis

A2780 and A2780/CP70 cells were treated in triplicates for 48 h using 7 concentrations of Dox and WFA both alone or combination of WFA/Dox at a constant ratio as described above. Viable cells were quantitated by MTT assays as described above and fraction affected was calculated from percent inhibition. Fraction affected was then used in CalcuSyn software to generate a dose-response curve and isobologram.

### Cell Apoptosis Assays using Flow Cytometry for Annexin V

A2780 were treated with Dox and WFA both alone or combination of WFA/Dox as described above for 24 h. Cells were dissociated with versene (Invitrogen), washed with PBS, and resuspended in Annexin V binding buffer to a concentration of 1×10^6^ cells/ml. Annexin V-FITC (2 µl, BD Biosciences) was incubated for 15 min in the dark with 100 µl of cell suspension. Four hundred µl of Annexin V binding buffer was added to the suspension. Two µl of propedium iodide (PI) was spiked into solution and immediately used on a FACSCaliber (BD Biosciences) as described by Betts et al [24]. Annexin V and PI stained cells were analyzed using FlowJo software.

### Reactive Oxygen Species (ROS) Assays

A2780 cell were seeded on glass bottom 35 mm^2^ dishes overnight followed by treatment with Dox and WFA as described above for 24 h. Cells were then incubated with 2 µM H_2_DCFDA (Invitrogen) in growth medium for 30 min at 37°C as described by Das et al [7]. Cells were washed with PBS, viewed under confocal microscope, and photographed.

### DNA Damage Analysis using TUNEL Assays

A2780 cells were seeded on chamber slides and treated with Dox and WFA as described above. Cells were then assayed for DNA damage using DeadEnd Fluorometric TUNEL assay kit (Promega) according to the manufacturer’s instructions. Cells were examined under confocal microscope and photographed.

TUNEL assays for tissue sections were performed using an ApopTag Plus Peroxidase Apoptosis Detection Kit (Millipore, Billerica, MA) according to manufacturer’s instructions.

### Protein Isolation and Western Blot Analysis

A2780 cells were seeded into 6-well plates and treated with Dox and WFA both alone or combination of WFA/Dox as described above for 24 h. Cell lysates were prepared as described previously [25]. Proteins were resolved on SDS-PAGE and transferred to a nitrocellulose membrane (GE Healthcare). Primary antibodies were diluted as indicated by the manufacturer and incubated overnight at 4°C. Antibody binding was revealed by peroxidase labeled secondary antibodies visualized using enhanced chemioluminescence (GE Healthcare) as described previously [26]. Blots were then re-probed with GAPDH to normalize differences in loading.

### Three Dimensional (3D) Tumor Growth Assays

A2780 cells (15,000/bead) were mixed with Hubiogel® in a ratio of 1∶4 and dispensed into 10 µl beads and allowed to polymerize before being suspended in warm growth medium to form spherical tumors. Each spherical tumor (1 mm^3^) were transferred to 96 well plate (one tumor/well) and treated with Dox (0.2, 2 µM), WFA (0.5, 2 µM), or combination of WFA/Dox (0.5 µM/0.2 µM or 2.0 µM/0.2 µM). Medium was replaced (twice/week) with medium containing fresh agent. Tumor growth was performed at day 1, 3, and 7 using MTT assays as described above. Tumors after treatment were incubated with calcein AM (Invitrogen) for 30 min and examined using Nikon B-2EIC fluorescence microscope using FITC filter block at Ex_465–495_ nm and Em_515–555_ nm) and photographed.

### Xenograft Tumor Formation and Treatment with Dox, WFA or WFA/DOX

A2780 cells were mixed with Matrigel (BD Biosciences) at a 1∶1 ratio. Cells (2×10^6^) were bilaterally injected subcutaneously into the ventral flank of 5–6 week old nu/nu mice (Charles River) and tumors were allowed to grow for 20 days until they reached to 100 mm^3^ in size as described previously [27]. The mice were then randomized into 6 groups and injected with: 1) PBS, 2) 10% DMSO +90% glyceryl trioctanoate (vehicle), 3) Dox 9 mg/kg, 4) Dox 1 mg/kg, 5) WFA 2 mg/kg, or 6) Dox 1 mg/kg + WFA 2 mg/kg. Mice were treated every other day i.p. using 100 µl volume and tumors were measured with digital caliper before each treatment. Dox was solubilized in saline whereas WFA was solubilized in DMSO and glyceryl trioctanoate (10∶90 v/v). Mice were sacrificed after 12 days of the start of treatment. All treatments were approved by IACUC, University of Louisville.

### Immunohistochemical Analysis of Tumor Tissues

Xenograft tumors were fixed in 10% formalin and embedded in paraffin for sectioning. Slides were deparaffinized in xylene and rehydrated in a graded series of ethanol. Antigen retrieval was conducted by incubating the slides in 10 mM sodium citrate, pH 6.0 for 20 min at 95°C followed by treatment with 0.3% H_2_O_2_ in methanol for 20 min [28]. Slides were processed using the Vectastain ABC Elite Anti-Rabbit kit (Vector Labs). Sections were incubated with primary antibodies for Ki67 (diluted 1∶50, Santa Cruz Biotechnology), CD31 (1∶50, AbCam), LC3B (1∶500, AbCam), and cleaved caspase 3 (1∶200, Cell Signaling) at 4°C overnight. Slides were rinsed with PBS and incubated with secondary antibody according to suppliers’ instructions. Color was developed using DAB (Vector Labs) and counterstained with hematoxylin QS (Vector Labs) to stain nuclei as described previously [28].

### Statistical Analysis

Values were expressed as mean ±SD. P values were determined by ANOVA analysis followed by Student-Newman-Keuls test for multiple comparisons.

## Results

### WFA Synergizes the Antitumor Effect of Doxorubicin

**Figure 1:**
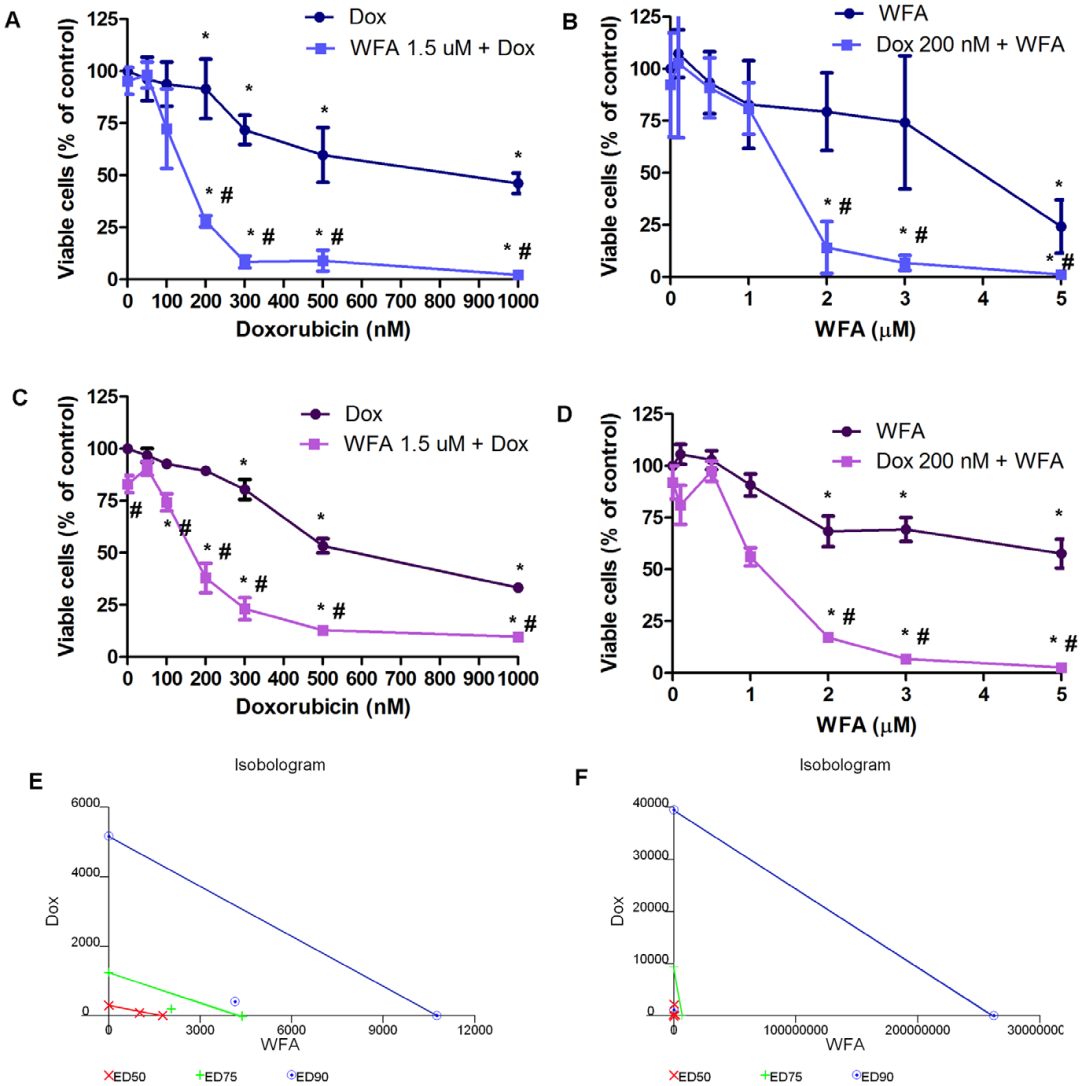
Cell proliferation of A2780 (A–B) and A2780/CP70 (C–D) cells on treatment with Dox and WFA, both alone or combination of WFA/DOX using MTT assays. A2780 and A2780/CP cells were plated into 96 well plates. After 24 h of plating, cells were treated with various concentrations of Dox and WFA, both alone or in combination. After 48 h of treatment, cell viability was assayed using MTT assays. Values shown are mean ±SD of four independent experiments. *P<0.05 compared to control, #p<0.05 compared to Dox or WFA alone. (E) Isobologram analysis of A2780 cells (n = 4) and (F) A2780/CP70 (n = 4) using 7 doses of Dox and WFA maintained at a constant ratio and cell death was assayed by MTT assays. Results were analyzed with CalcuSyn software.

Dox is typically used at 5 µM to mimic the concentration found in plasma of patients undergoing Dox treatment [29]. However, at this dose, patients present with serious side effects since a concentration of 1 µM is required to maintain various mechanisms of actions of Dox [29]. To minimize or eliminate these side effects, we explored the possibility of using a Dox/WFA combination treatment. Ovarian cancer cell lines A2780 and CAOV3 and a cisplatin-resistant cell line A2780/CP70 were treated with various concentrations of Dox and WFA both alone and in combination. Dox/WFA combination inhibited cell proliferation of all three cell lines in a dose- and time-dependent manner. When Dox and WFA were used alone, the IC_50_ values for A2780 cells after 48 h of treatment were 0.8 µM and 4.1 µM respectively (Fig. 1A–B). When cells were co-treated with a combination of Dox with 1.5 µM of WFA, the IC_50_ value for Dox decreased to 0.16 µM (Fig. 1A). Similarly when 200 nM of Dox was combined with WFA, the IC_50_ value for WFA decreased to 1.5 µM (Fig. 1B). Cells when co-treated with 200 nM of Dox and 2.0 µM of WFA resulted in 90 to 95% cell death (Fig. 1B), whereas treatment of cells with Dox alone (200 nM) and WFA alone (2.0 µM) resulted in 9% and 20% inhibition respectively. For A2780/CP70 cells, the IC_50_ values for Dox and WFA were 0.65 µM and 6 µM respectively. Combining Dox with 1.5 µM of WFA reduced the IC_50_ value of Dox to 0.18 µM, and combining WFA with 200 nM of Dox reduced the IC_50_ value to 1.2 µM (Fig. 1C–D). CAOV3 cells were more sensitive to treatment with Dox and WFA alone or combination of Dox/WFA (results not shown). IC_50_ values are summarized in Table 1. These results suggest that the Dox/WFA combination works in a synergetic manner to mediate antitumor activity. Cell proliferation data after 24 h and 72 h of treatment are shown in Fig. S1 and S2.

**Table 1:** IC_50_ values of doxorubicin, Withaferin A, on treatment of A2780, A2780/CP70 and CAOV3 cells for 48 h with DOX and WFA, both alone or combination of DOX and WFA.

	IC_50_ (µM) values		
Compounds	A2780	A2780/CP70	CAOV3
Dox	0.8	0.65	0.30
WFA 1.5 µM + Dox	0.16	0.18	0.05
WFA	4.1	6.0	1.0
Dox 200 nM + WFA	1.5	1.2	0.70

To confirm that the effect of combination of WFA with Dox was synergistic, we performed isobologram analysis. Both A2780 and A2780/CP70 cells were treated with 7 concentrations of Dox and WFA in a constant ratio for 48 h and cell proliferation was analyzed by MTT assays. CalcuSyn software was used to generate the isobolograms, demonstrating that Dox and WFA act synergistically (Fig. 1E–F) for both the cell lines.

To determine if apoptosis was the cause of cell death, we performed Annexin V-FITC flow cytometry in A2780 cells treated with Dox and WFA both alone or in combination. Analysis of Dox, WFA, and Dox with WFA treated samples showed a non-significant increase over control for Annexin V (Fig. S3). In order to confirm our technique, positive control samples were produced using UV exposure for 30 sec and analyzing cells 4 h, 6 h, and 24 h after exposure to ensure efficiency of staining (Fig. S4). In addition, we investigated intrinsic apoptotic proteins phospho-BAD^136^ (pBAD^136^) and Bcl-xL. We found no significant changes in pBAD^136^ or Bcl-xL (Fig. S5), indicating that an alternative pathway to intrinsic apoptosis is being used to induce cell death.

### Dox and WFA Produce ROS to Induce Cell Death

**Figure 2:**
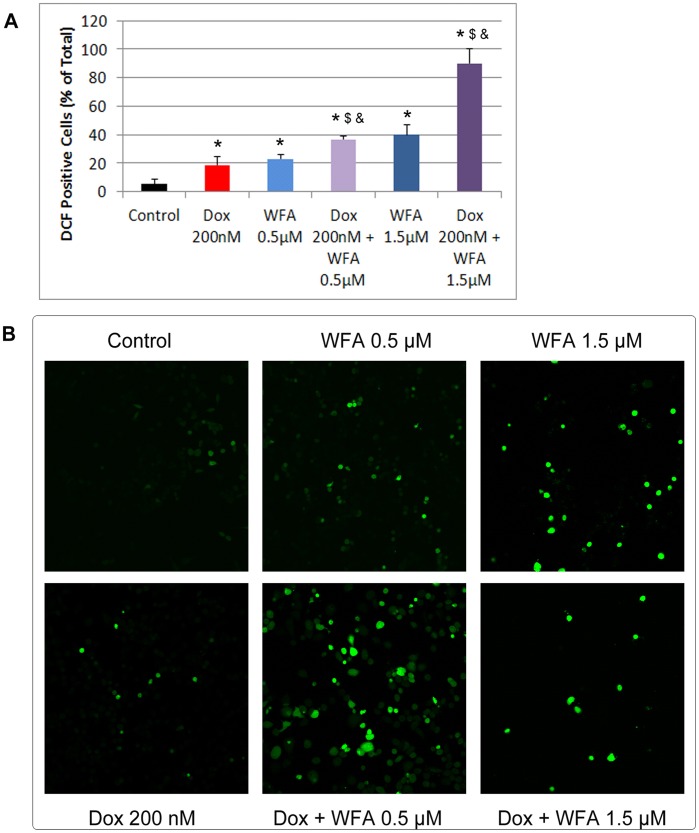
ROS generation in A2780 cells. A2780 cells were plated into glass bottom dishes. After 24 h of plating, cells were treated with Dox and WFA, both alone or combination of WFA/DOX as described in Figure 1. After 24 h of treatment, medium was replaced with fresh medium containing H_2_DCFDA and incubated for 30 min. Cells were rinsed with PBS and examined under confocal microscope. (A) ROS positive cells (green color) were counted based on 3 low power fields. Mean ±SD. P values were determined by ANOVA analysis followed by Student-Newman-Keuls test for multiple comparisons. *P<0.05 from control, $P<0.05 compared to Dox, &P<0.05 compared to WFA. (B) Confocal microscopy analysis of cells indicating generation of ROS (green color).

Dox is known to produce ROS as a part of its mechanisms [4], [5]. There have also been numerous reports about WFA generating ROS production as one part of its apoptotic mechanisms in various cancer types [12], [17], [18], [30]. Therefore, we asked whether WFA could enhance the effect of low concentration of Dox after 24 h of treatment, we used H_2_DCFDA to determine generation of ROS. H_2_DCFDA is a stable non-polar compound that is readily diffused into the cells. This compound is then hydrolyzed by intracellular esterases to form DCFH, which in turn is oxidized by hydrogen peroxide to yield the highly fluorescent compound 2′7′-dichlorofluorescein (DCF). After 6 h of treatment with WFA 1.5 µM significantly increased ROS positive cells from 2% to 17% compared to control cells (results not shown). After 24 h of treatment, Dox 200 nM showed a low number of ROS positive cells, 18% (Fig. 2A–B). While WFA 0.5 µM (23%) was not significantly different from Dox, combination of Dox 200 nM with WFA 0.5 µM resulted in a significant increase to 37%. This effect was greatly enhanced with a combination of Dox 200 nM with WFA 1.5 µM, increasing to 90% ROS positive cells (Fig. 2A–B). Treatment with WFA 2 µM damaged the cells too severely to produce ROS, indicating that the effect of WFA on ROS production is dose-dependent and upon combination with Dox elicits a synergistic effect.

**Figure 3:**
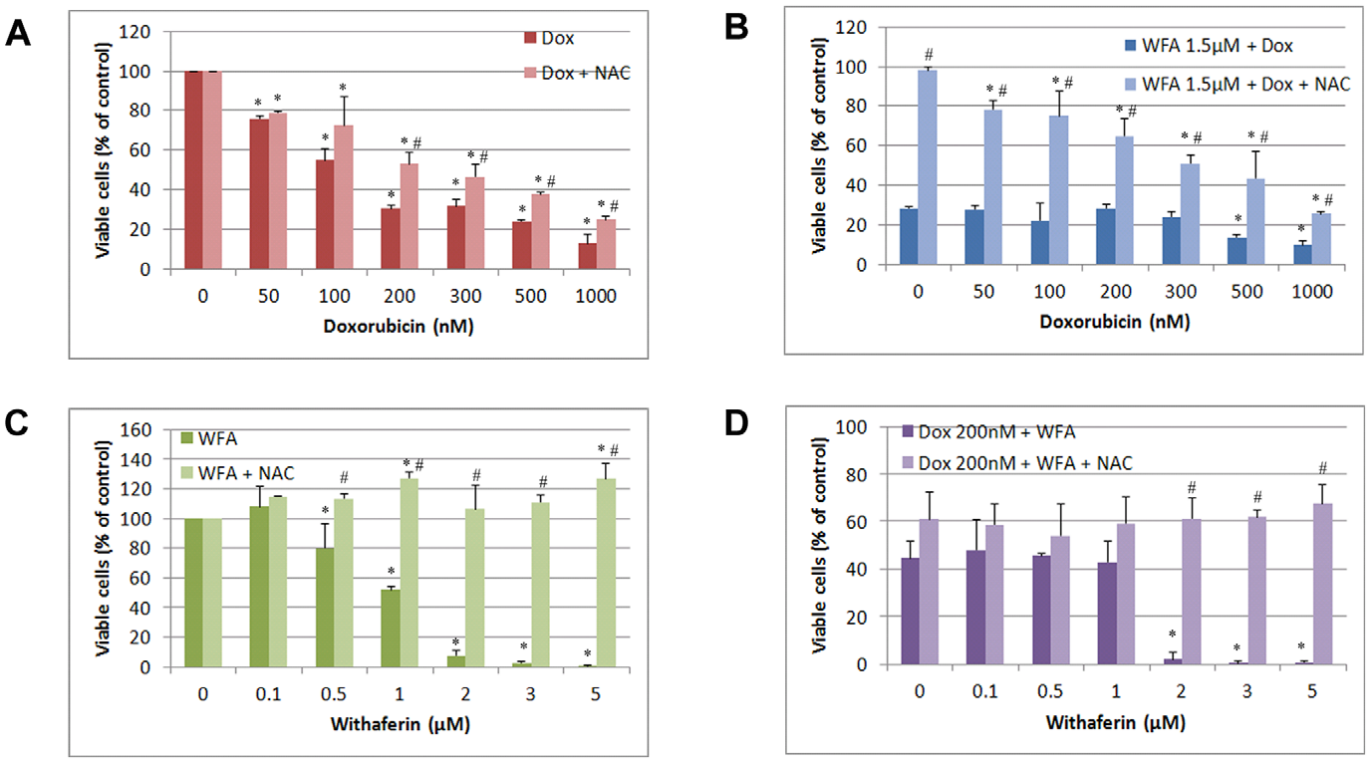
Effect of non-enzymatic ROS antioxidant NAC on A2780 cell proliferation after 48 h of treatment. A2780 cells were co-treated with NAC and Dox, WFA or combination of WFA/WFA for 48 h. Cell proliferation was determined using MTT assays. Values shown are mean ±SD of three independent experiments. P<0.05 compared to control, #P<0.05 compared to no NAC and NAC.

**Figure 4:**
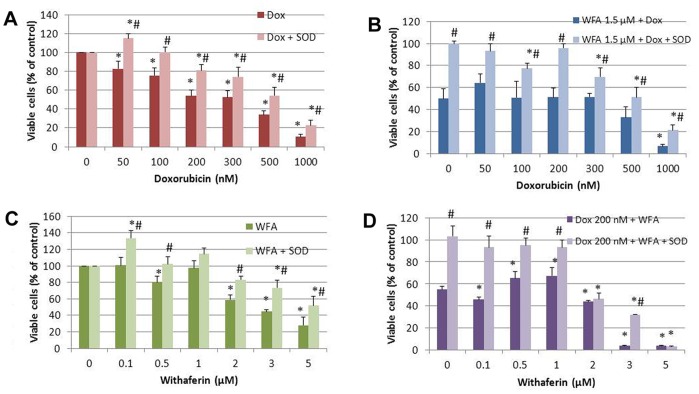
Effect of enzymatic antioxidant SOD (100 units/ml) on A2780 cell proliferation after 48 h of treatment. A2780 cells were co-treated with SOD and Dox, WFA or combination of WFA/Dox for 48 h. Cell proliferation was determined using MTT assays. Values shown are mean ±SD of three independent experiments. *P<0.05 compared to control, #P<0.05 compared to no SOD and SOD.

To confirm that ROS are responsible for our observed cell death, we co-treated A2780 cells with the ROS scavenger N-acetyl-L-cysteine (NAC, 5 mM) or with enzymatic antioxidants superoxide dismutase (SOD) and catalase along with Dox and WFA treatments for 24 and 48 h as described above. While NAC was ineffective to block cell death induced by Dox at 24 h, it provided moderate protection after 48 h of treatment (Fig. 3A) determined by MTT assays. NAC was highly effective to block cell death induced by WFA after 24 h and continued to provide protection after 48 h of incubation (Fig. 3C). NAC was also effective in blocking Dox/WFA combination treatment (Fig. 3B–D). To differentiate between the major form of ROS produced by Dox and WFA treatment, we treated the cells with enzymatic antioxidants catalase 500 U/ml or SOD 100 U/ml. It has been reported that catalase is frequently used by cells to degrade hydrogen peroxide [31] while SOD specifically catalyzes superoxide anions [32]. After 48 h of treatment, SOD significantly blocked cell death induced by Dox and WFA alone and in combination (Fig. 4). Similar to SOD, catalase showed protection of cell death by Dox and WFA both alone or combination of WFA/Dox (results not shown), indicating that superoxide anions are the major ROS species produced by Dox and WFA.

**Figure 5:**
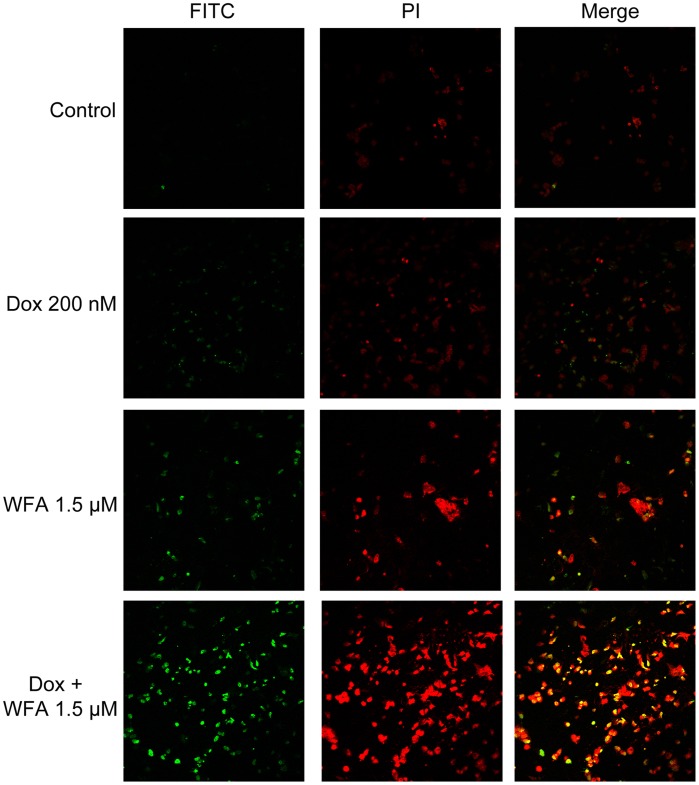
DNA damage (TUNEL) assay of A2780 cells after 24 h of treatment. A2780 cells were treated with Dox, WFA both alone or combination of WFA/Dox. After 24 h of treatment, DNA damage was analyzed using TUNNEL assays. Images were obtained using confocal microscopy at 20X magnification.

As ROS causes DNA damage, we performed TUNEL assay to visualize the extent of DNA damage when treated with Dox and WFA alone or in combination. After 24 h of treatment, Dox 200 nM resulted in DNA damage in few cells while WFA 1.5 µM alone slightly increased number of damaged cells (Fig. 5). However, treatment with Dox 200 nM and WFA 1.5 µM combination resulted in an enhanced effect to induce DNA damage. Nearly every cell showed DNA damage (Fig. 5).

### Combination of Dox and WFA Induced Cell Death by Autophagy

**Figure 6:**
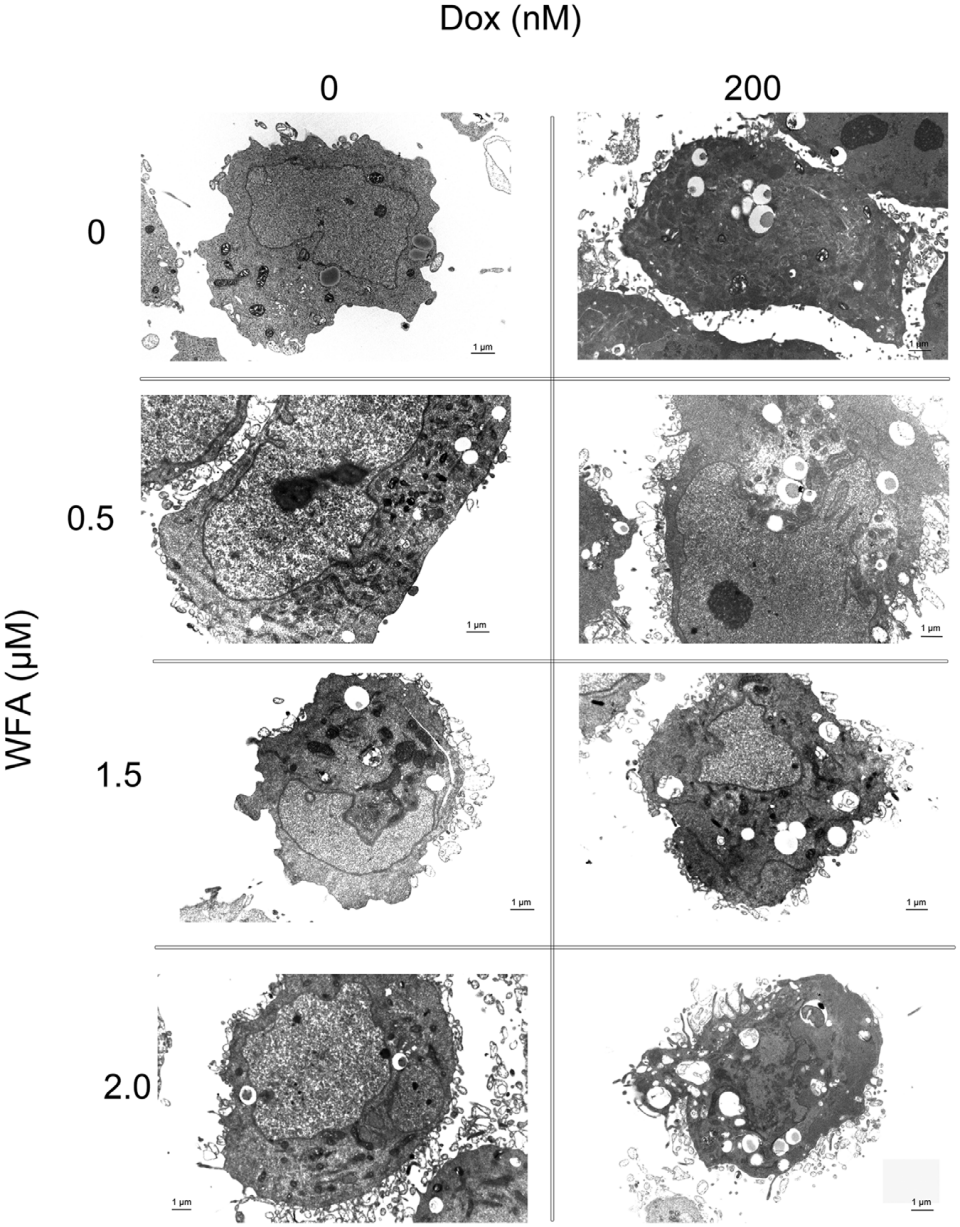
Analysis of autophagy using transmission electron microscope (TEM). A2780 cells were treated with Dox and WFA, both alone or combination of WFA/Dox. After 24 h of treatment, cells were rinsed with PBS, fixed and processed for TEM analysis. Electron microscopic images at 5,600X magnification are shown.

Various anticancer chemotherapies including Dox have been shown to induce autophagy, which cooperates with apoptosis to induce cell death [33]–[38] as a means to eliminate damaged organelles that may produce a high level of ROS and hence limit chromosomal instability [39]. Therefore investigating the role of chemotherapy agent Dox combined with WFA in autophagy is an avenue of interest. Electron microscopy analysis of control cells treated with DMSO showed the presence of mitochondria and an intact nuclear envelop (Fig. 6). While WFA 0.5 µM alone had little or no effect, WFA 1.5 and 2 µM showed few autophagosomes as an indicator of autophagy, but left the mitochondria intact, possibly as an adaptation mechanism. Cells when treated with Dox 200 nM alone showed formation of autophagosomes containing cytoplasm and destruction of the mitochondria. Treatment of cells with Dox/WFA combination resulted in an enhanced effect in a dose-dependent manner. Dox 200 nM plus WFA 2 µM showed intense autophagic vacuoles along with collapse of the nuclear envelop, membrane disintegration, and absence of mitochondria (Fig. 6), indicating intense cell damage upon treating with Dox/WFA combination.

**Figure 7:**
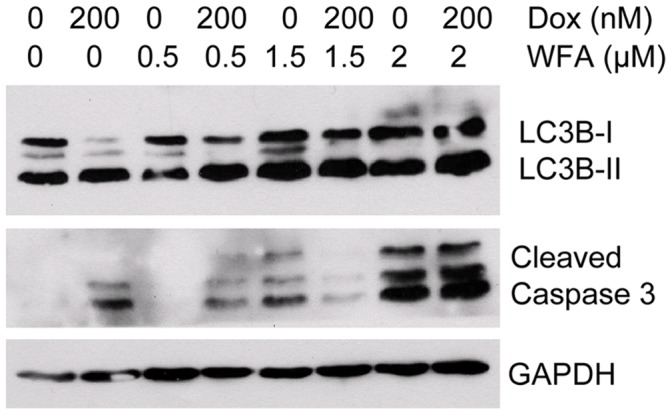
Western blot analysis of autophagy pathway of A2780 cells treated for 24 hr. A2780 cells treated with Dox and WFA, both alone or combination of WFA/Dox. After 24 h of treatment, cells were washed with PBS and lysed. Western blot analysis was performed for LC3B, Caspase 3 and GAPDH proteins.

To confirm the induction of autophagy upon treatment of cells with Dox/WFA combination, we determined the expression of the canonical marker of autophagosome formation, microtubule-associated protein-1 light chain 3B (LC3B) [38]. Western blot analysis of the cells showed two specific bands: an upper band (18 kDa) representing LC3B-I and a lower band (16 kDa) corresponding to LC3B-II (Fig. 7). Cytosolic LC3B-I is converted to LC3B-II through lipidation and allows LC3B-II to become associated with autophagic vesicles. Treatment with Dox induced production of LC3B-II (Fig. 7), while WFA alone stimulated production of the pre-cursor LC3B-I as well as LC3B-II (Fig. 7). Combination treatment enhanced LC3B-II in a dose-dependent manner with Dox 200 nM with WFA 2 µM showing the highest expression (Fig. 7). To determine if autophagy was an adaptation response or a mechanism of cell death, we investigated cleaved caspase 3 as a marker for cell death. Western blot analysis showed a modest increase in cell treated with Dox 200 nM. In contrast, WFA at 0.5 µM showed no indication of cell death, while WFA 1.5 and 2 µM showed an increase in the level of cleaved caspase 3. Treatment of cells with Dox/WFA combination showed a further enhancement of cell death in a dose-dependent manner (Fig. 7), indicating that autophagy is promoting cell death rather than inducing an adaptation mechanism to promote cell survival with Dox/WFA combination treatment.

### Effect of Dox and WFA on 3D Tumors in vitro

**Figure 8:**
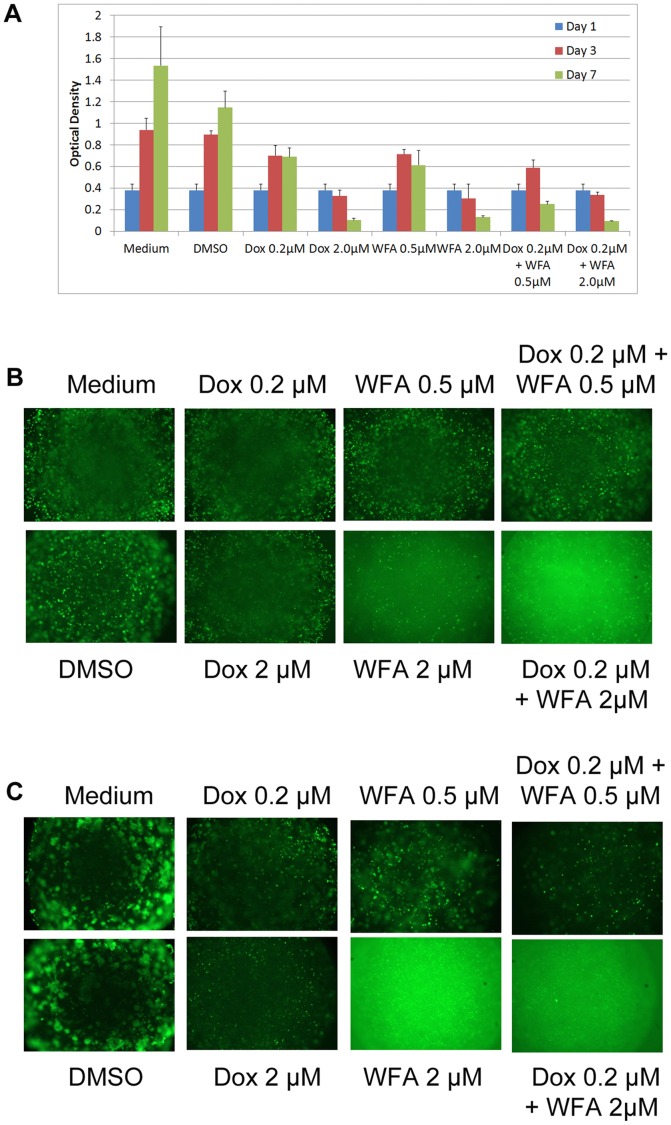
In vitro analysis of tumor growth using 3D tumor model. (A) A2780 Cells were combined with Hubiogel® in a 1∶4 ratio and grown from 10 µL beads. Tumors were treated with Dox and WFA, both alone or combination of WFA/Dox. Tumors were treated twice/week by replacing the medium with medium containing fresh agent. Tumor growth was measured using MTT assays after day 3 or 7 of treatment. (B–C) Tumors after treatment were incubated with calcein AM for 30 min, and images were taken using fluorescence microscope after 3 days of treatment (B) or 7 days of treatment (C).

In addition to assaying inhibition of tumor cell growth, we evaluated the effects of Dox and WFA both alone or WFA/Dox combination for their anti-tumor efficacy using a 3D mini-tumor model that emulates *in vivo*-like multicellular tumor growth and biology. Viable mini-tumors (1 mm^3^ size) of A2780 ovarian cancer cells were generated using a 3D human biogel culture system (HuBiogel®, Vivo Biosciences Inc.). Hubiogel® has been shown to represent the human matrix more accurately than Matrigel in order to predict preclinical endpoints [40]. Mini-tumors were treated with 1) Dox 0.2 µM, 2) Dox 2.0 µM, 3) WFA 0.5 µM, 4) WFA 2.0 µM, 5) Dox 0.2 µM with WFA 0.5 µM, and 6) Dox 0.2 µM with WFA 2 µM. Measurements of tumor growth were performed at day 1, 3, and 7 using MTT assays and fluorescence microscopy. Medium and DMSO treated tumors continued to grow throughout treatment, whereas Dox 0.2 µM had their growth halted at day 7 (Fig. 8A). Dox 2.0 µM alone and WFA 2.0 µM alone treated tumors showed reduced growth and this inhibitory effect was enhanced upon treatment with Dox 0.2 µM plus WFA 2.0 µM (Fig. 8A). Combination of Dox 0.2 µM with WFA 0.5 µM achieved a drastically enhanced effect compared to either compound alone (Fig. 8A). Microscopy analysis of tumors after day 3 and 7 is shown in Fig. 8B and 8C respectively, indicating synergetic effect of Dox and WFA combination on suppression of tumor growth.

### Effect of Dox and WFA on Xenograft Tumor Growth

**Figure 9:**
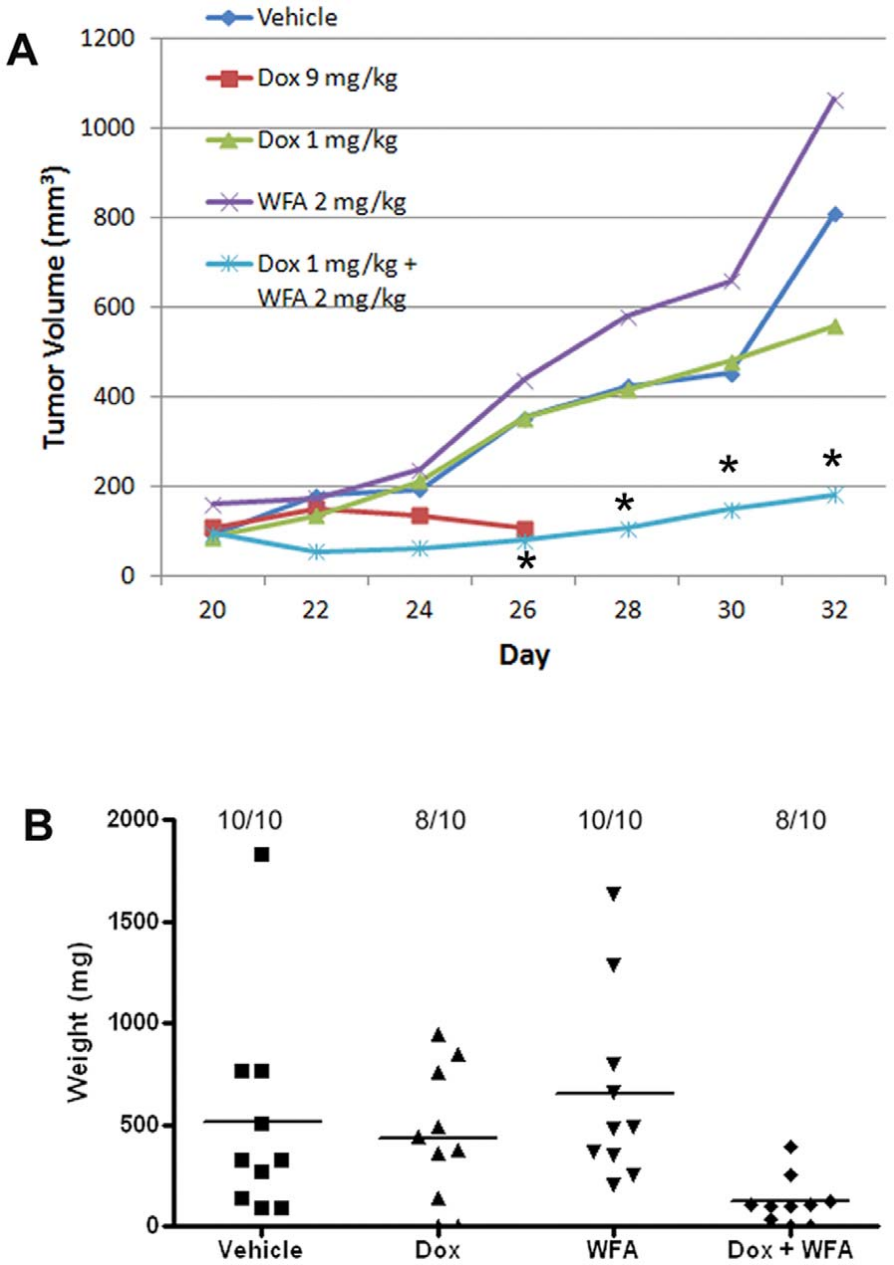
S.C. tumors were generated in nude mice and treated with PBS, Vehicle, Dox 1 mg/kg, Dox 9 mg/kg, WFA 2 mg/kg or Dox 1 mg/kg plus WFA 2 mg/kg. (A) Tumors growth was measured from day 20–32 (post-cell injection) and treated every other day *P<0.05. (B) Tumor weight at day 32 collected immediately after sacrificing the animals.

To study the effect of Dox and WFA alone or in combination on tumor growth *in vivo*, mouse tumor xenografts were developed by injecting A2780 cells (2×10^6^) subcutaneously bilaterally in the ventral flank of 5–6 week old nu/nu mice. Tumors were allowed to grow until they reached 100 mm^3^ in size. At day 20 of post-cell injection, mice were randomized into 6 groups of 5 mice each and treated with different agents: 1) negative control (PBS), 2) vehicle control (10% DMSO and 90% glyceryl trioctanoate), 3) Dox 9 mg/kg, 4) Dox 1 mg/kg, 5) WFA 2 mg/kg, and 6) Dox 1 mg/kg with WFA 2 mg/kg as described in materials and methods. Tumors were measured every other day and mice were administered with 100 µl i.p. volume for 12 days for a total period of 32 days. Mice receiving Dox 9 mg/kg appeared to be very sick with a loss of appetite resulting in weight loss after the first treatment and subsequently died after 4 treatments. Mice in the other groups appeared to be healthy with no loss of appetite or weight during the entire treatment period. The tumor volume was not significantly different between vehicle, Dox 1 mg/kg and WFA 2 mg/kg groups. However, mice receiving Dox 1 mg/kg with WFA 2 mg/kg (group 6) showed a highly significant (70 to 80%) reduction in tumor growth (Fig. 9A). Similarly, tumor weight measured at day 32 collected at the time of sacrificing the animals, showed a drastic decrease in the Dox 1 mg/kg with WFA 2 mg/kg group (Fig. 9B) compared to other groups indicating that combination of WFA with Dox elicits a synergistic effect on tumor suppression of tumor growth *in vivo*.

**Figure 10:**
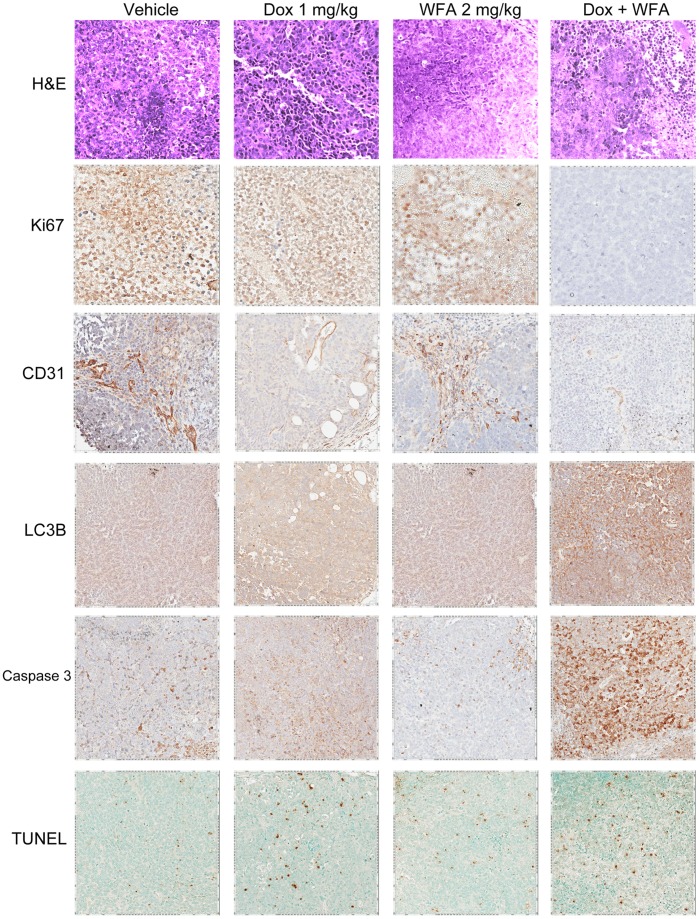
Immunohistochemistry compilation of tumor tissues developed with DAB (brown) and counterstained with hematoxylin to stain nuclei (blue). Negative control samples were tissues without primary antibody. TUNEL assay was performed using ApopTag Plus Peroxidase Apoptosis Detection Kit.

H&E analysis of the xenograft tumor sections identified the tumors as serous adenocarcinoma (Fig. 10). Vehicle group tumors were high grade with extensive necrosis. Dox 1 mg/kg (group 4) also had extensive necrosis. However, WFA 2 mg/kg (group 5) and Dox 1 mg/kg with WFA 2 mg/kg (group 6) were poorly differentiated with tumor necrosis. Immunohistochemistry for proliferation marker Ki67 showed intense staining in the vehicle group with less intense staining in Dox 1 mg/kg (group 4) and WFA 2 mg/kg (group 5). Dox 1 mg/kg with WFA 2 mg/kg (group 6) showed no or undetectable staining for Ki67, suggesting that combination therapy effectively reduced tumor growth (Fig. 10). Staining of sections with microvessel marker CD31 showed a high amount of microvessel formation in tumors collected from vehicle treated mice, which was reduced in Dox 1 mg/kg (group 4) and WFA 2 mg/kg (group 5). Dox 1 mg/kg with WFA 2 mg/kg (group 6) further reduced the amount of CD31 staining (Fig. 10).

We also performed immunohistochemistry for autophagy marker LC3B to validate the mechanism of action we observed *in vitro*. Tumors collected from animals that received vehicle control or WFA 2 mg/kg showed a low amount of positive cells, whereas animals treated with Dox 1 mg/kg showed a moderate level of expression. This was further enhanced with combination treatment (group 6, Fig. 10), demonstrating that combination therapy lead to the induction of autophagy. Staining of tumor sections for cleaved caspase 3 showed a low level of staining in vehicle and WFA 2 mg/kg treated groups. Cleaved caspase 3 was increased in Dox 1 mg/kg which was synergistically enhanced in Dox 1 mg/kg with WFA 2 mg/kg treated group (Fig. 10).

TUNEL assays of tumors revealed DNA damage in tumors collected from animals receiving Dox 1 mg/kg with a lower amount in WFA 2 mg/kg (Fig. 10). However, combination of Dox 1 mg/kg with WFA 2 mg/kg showed enhanced DNA damage compared to WFA and Dox alone (Fig. 10), indicating an enhanced effect with the combination of Dox with WFA in the induction of DNA damage.

## Discussion

Dox or its liposomal preparation, Doxil has been used in combination with several compounds for various cancer types. Doxil used in combination with bevacizumab in patients with recurrent ovarian cancer achieved a 33% response rate [41]. Doxorubicin has been combined with other compounds, including chebulagic acid [42] and arsenic trioxide [43] in hepatocellular carcinoma cell lines, with sildenafil in prostate cancer cell lines PC-3 and DU145 [7], and with a synthetic analog of curcumin HO-3867 in breast cancer cell line MCF-7 [8]. Combination therapy has been shown to achieve a complementary outcome with Dox to increase cancer cell toxicity without myocardial toxicity [7], [8].

There has been increasing support for anticancer drugs from natural products, drawing on Chinese, Kampo, and Ayurvedic medicine for promising compounds such as WFA. The cytotoxic activity of WFA has been established with IC_50_ value of approximately 5 µM after 72 h in a panel of cancer cell lines and a transformed fibroblast cell line [44], however this did not include an ovarian cancer cell line. In our study using cisplatin sensitive ovarian cancer cell line A2780, cisplatin-resistant ovarian cancer cell line A2780/CP70, and ovarian cancer cell line that expresses a mutant form of p53 gene CAOV3, we showed the IC_50_ values for WFA were 4.1, 6, and 1 µM respectively after 48 h of treatment. With the addition of Dox 200 nM, the IC_50_ values were reduced to 1.5, 1.2, 0.7 µM respectively (Fig. 1). Isobologram analysis showed synergistic interaction between Dox and WFA using CalcuSyn software analysis (Fig. 1E–F). WFA has been shown to reduce *in vivo* tumor growth of human pancreatic [15] and breast cancer cells [9] at a dose of 6 mg/kg and 4 mg/kg respectively. In our study we showed that a low dose of WFA (2 mg/kg) alone or Dox (1 mg/kg) alone was ineffective in suppressing tumor growth *in vivo*. However, combining a suboptimal dose of WFA with a low dose of Dox (1 mg/kg) showed a significant (70 to 80%) suppression of tumor growth (Fig. 9A).

Apoptosis is considered as the principle mechanism by which chemotherapy agents induce cancer cell death. It is a highly conserved cellular program that eliminates damaged and infected cells. It consists of two major pathways: the extrinsic pathway that is mediated by death receptors and the intrinsic pathway that is mediated by the mitochondria. Both pathways lead to activation of caspases, cysteine proteases that cleave different substrates resulting in cellular breakdown [31]. However, more recent evidence suggests that anticancer agents also induce other forms of non-apoptotic cell death including necrosis, mitotic catastrophe, autophagy, and senescence [45]. Various anticancer chemotherapies including Dox have been shown to induce autophagy which cooperates with apoptosis to induce cell death [33]–[38]. However, autophagy enables cells to survive harsh conditions such as chemotherapy treatment and thus conferring resistance [33]. As such, it is still unclear why autophagy participates in cell death in some instances while preventing it in others, especially since both effects can be observed with the same anticancer compound. It has been suggested that as the level of autophagy increases the likelihood of the induction of cell death rather than survival [33]. In addition, autophagy can have tumor suppressive functions. One proposed pathway suggests that autophagy eliminates damaged organelles that may produce high levels of ROS and therefore limit chromosomal instability [39]. We found that treatment with Dox in combination with WFA increased ROS production as early as 6 h of treatment and continued to increase by 24 h of treatment (Fig. 2). Consistent with previous reports on Dox and WFA [4], [5], [12], [17], [18], [30], we confirm that both agents produce ROS, although ROS was greater in WFA treated cells (Fig. 2). Combination of Dox with WFA further enhanced ROS production (Fig. 2). Blocking of ROS production by NAC showed a complete remission of cell death in WFA treated cells and Dox with WFA treated cells (Fig. 3), suggesting that ROS production as the major mechanism of inducing cell death for WFA. Furthermore, treating the cells with SOD lead us to determine that superoxide anions were the major ROS species produced, especially in the case of Dox (Fig. 4). As SOD treatment was not sufficient completely in blocking the cell death compared to NAC in WFA treated cells, it is likely that WFA produces more than one species of ROS during cellular processing. ROS-mediated autophagy has been observed in a number of different carcinoma cell lines [46]–[48]. Additionally, blocking of ROS production with ROS scavengers and antioxidants reduced autophagic cell death in various solid tumors cell lines [48], [49]. Mitochondrial ROS damage the mitochondrial membrane and result in leakage of ROS to the cytosol where they can damage other organelles as well as cause DNA damage and oxidation of amino acids and polydesaturated fatty acids [50], [51]. As a result of ROS production, we performed the TUNEL assay to assess DNA damage. We showed that Dox alone slightly caused DNA damage with a greater increase with WFA 1.5 µM treated cells (Fig. 5). However, combining Dox with WFA resulted in a significant amount of DNA damage in nearly all cells (Fig. 5). Electron microscopy analysis revealed the presence of autophagic vacuoles (Fig. 6) which was confirmed with Western blot by analysis of LC3B-II (Fig. 7). As a means to determine if autophagy was participating in cell survival or cooperating with apoptosis to induce cell death, we analyzed cleaved caspase 3 levels by Western blot and showed that Dox slightly increased caspase 3 with an enhanced effect with the addition of WFA (Fig. 7). However, we observed no change in the level of Bcl-xL, pBAD^136^, or Annexin-V flow cytometry (Fig. S3 and S5). Annexin V protein has a strong affinity for phosphatidylserine, which is translocated from the inner leaflet of the cellular membrane to the outer leaflet during the early events of apoptosis [52]. However, Annexin V staining precedes the loss of membrane integrity, which accompanies the late stages of cell death resulting from either apoptotic or necrotic processes. It is possible that Dox damaged the cellular membrane and thus prevented staining of Annexin V. Taken together our results suggest that ROS production lead to the induction of autophagy, and DNA damage, leading to the activation of caspase 3 to induce apoptosis.

As cells grown in monolayer respond differently than cells growing as spheres, we used two different tumor models to investigate the therapeutic effects of Dox and WFA both alone or in combination. The first was an *in vitro* 3D tumor model generated using a biologically-active human extracellular matrix, HuBiogel®. The major components of HuBiogel® are collagen type I and IV, laminin, entactin, tenascin, and heparan sulfate proteoglycan [53]. Unlike Matrigel that is based on a reconstituted mouse matrix and contains mitogenic factors while lacking stromal components that affect not only tumor growth but response to drug treatment, HuBiogel® allows host cells to grow, organize, and function as mini-tissues [40]. In addition, because, it is human in origin, it allows for a better prediction of patient response to drug treatment [40]. Using A2780 cells to generate 3D tumors, we showed tumor regression in response to Dox and WFA after 3 or 7 days of treatment (Fig. 8A–B). The second tumor model was the classic *in vivo* xenograft tumor model in nu/nu mice using A2780 cells. We demonstrated that a low dose of Dox (1 mg/kg) combined with a suboptimal dose of WFA (2 mg/kg) was highly effective in suppressing tumor progression (Fig. 9) by reducing proliferation and angiogenesis while increasing autophagy, DNA damage, and apoptosis (Fig. 10), indicating that combining WFA with Dox reduces the dosage requirement of Dox to suppress tumor growth, and hence could minimize or eliminate the side effects including myocardial toxicity associated with high doses of Dox used to treat various solid cancers including ovarian cancer.

### Conclusion

**Figure 11:**
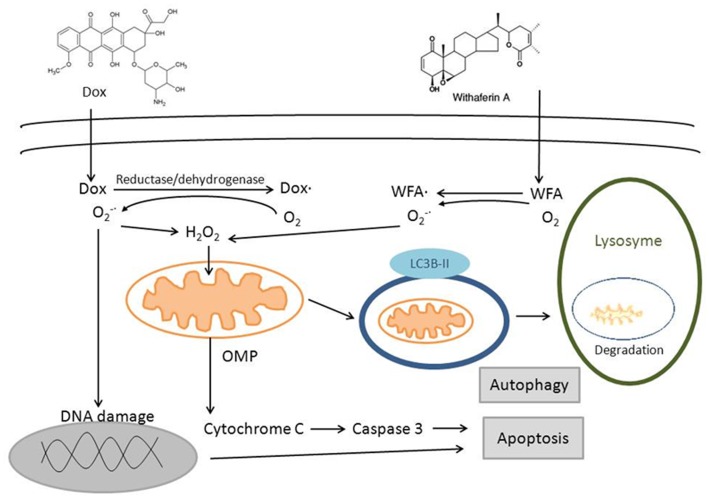
Schematic summary of mechanisms of cell death induced by Dox/WFA combination treatment.

In our present study, combination of Dox with WFA resulted in cell death through ROS-mediated autophagy (LC3B, caspase 3) and DNA damage (Fig. 11). We have also demonstrated the synergistic effect of Dox/WFA combination on ovarian cancer cell death and suppression of tumor growth *in vitro* using a 3D tumor growth model and *in vivo* using a xenograft tumor model. Our results suggest that combining low dose of Dox with suboptimal dose of WFA can serve as a potential combination therapy for the treatment of ovarian cancer with the potential to minimize/eliminate the side effects associated with high doses of Dox. In addition, WFA/Dox combination may be applicable for cisplatin-sensitive as well as cisplatin-resistant ovarian cancers.

## Supporting Information

Figure S1 **Cell proliferation analysis using MTT assays for ovarian cancer cell lines. A2780 cell line (A–B) (n = 4), A2780/CP70 (C–D) (n = 4) after 24 h of treatment.** *P<0.05 compared to control, #p<0.05 compared to Dox or WFA alone.
(TIF)

Figure S2 **Cell proliferation analysis using MTT assays for ovarian cancer cell lines. A2780 (A–B) (n = 4), A2780/CP70 (C–D) (n = 4) after 72 h of treatment.** *P<0.05 compared to control, #p<0.05 compared to Dox or WFA alone.
(TIF)

Figure S3 **Flow cytometry for Annexin V-FITC for cells treated with Dox and WFA both alone or in combination.** A2780 cells were treated with Dox and WFA both alone or combination of WFA/Dox for 24 h as described in Figure 1, dissociated with versene, and stained with Annexin V-FITC and PI. Samples were run on a FACSCaliber and analysis was performed with FlowJo software.
(TIF)

Figure S4 **Flow cytometry for Annexin V-FITC for UV treated cells.** A2780 cells were exposed to UV for 30 sec and harvested with versene after indicated time and then stained with Annexin V-FITC and PI. Samples were run on a FACSCaliber and analysis performed with FlowJo software.
(TIF)

Figure S5 **Western blot analysis of intrinsic apoptosis proteins of A2780 cells treated for 24 hr.**
(TIF)
