# Supplementary figures and images for: Withaferin A Synergizes the Therapeutic Effect of Doxorubicin through ROS-Mediated Autophagy in Ovarian Cancer
Source: PLoS One. 2012 Jul 30;7(7):e42265. doi: 10.1371/journal.pone.0042265 (PMC3408484; doi:10.1371/journal.pone.0042265)

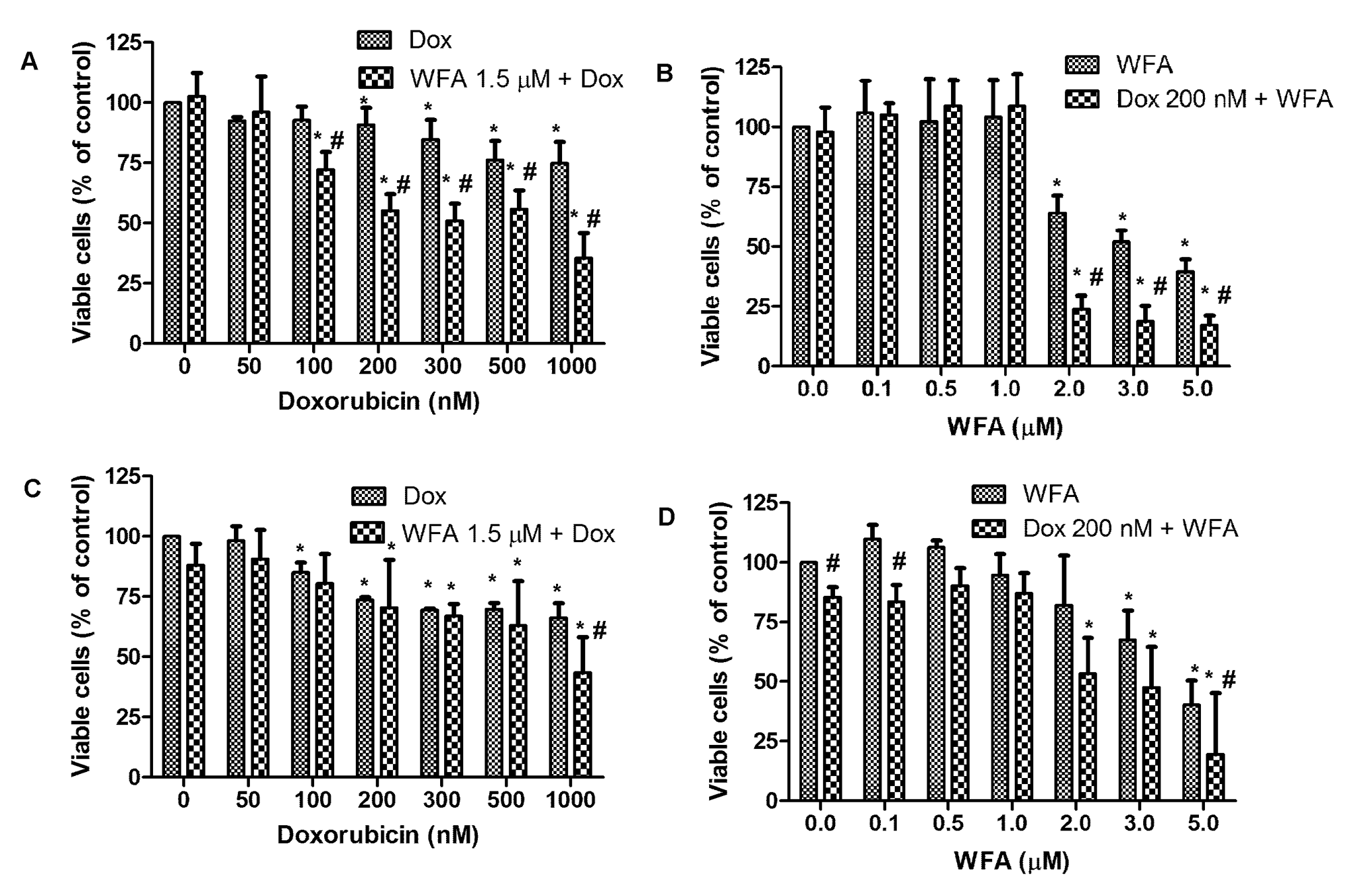

Supplement: Figure S1 — Cell proliferation analysis using MTT assays for ovarian cancer cell lines. A2780 cell line (A–B) (n = 4), A2780/CP70 (C–D) (n = 4) after 24 h of treatment. *P<0.05 compared to control, #p<0.05 compared to Dox or WFA alone. (TIF) [file pone.0042265.s001.tif]

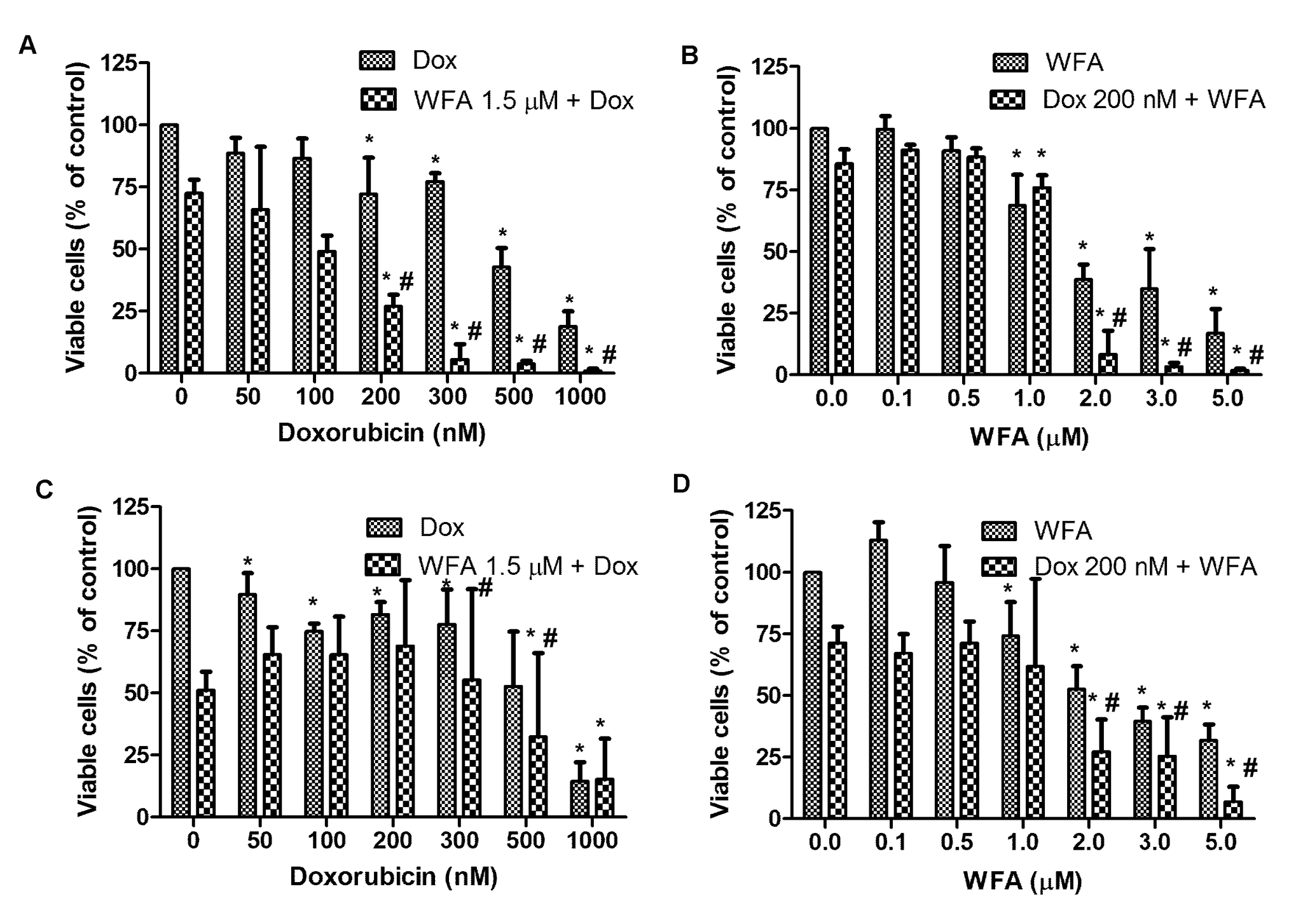

Supplement: Figure S2 — Cell proliferation analysis using MTT assays for ovarian cancer cell lines. A2780 (A–B) (n = 4), A2780/CP70 (C–D) (n = 4) after 72 h of treatment. *P<0.05 compared to control, #p<0.05 compared to Dox or WFA alone. (TIF) [file pone.0042265.s002.tif]

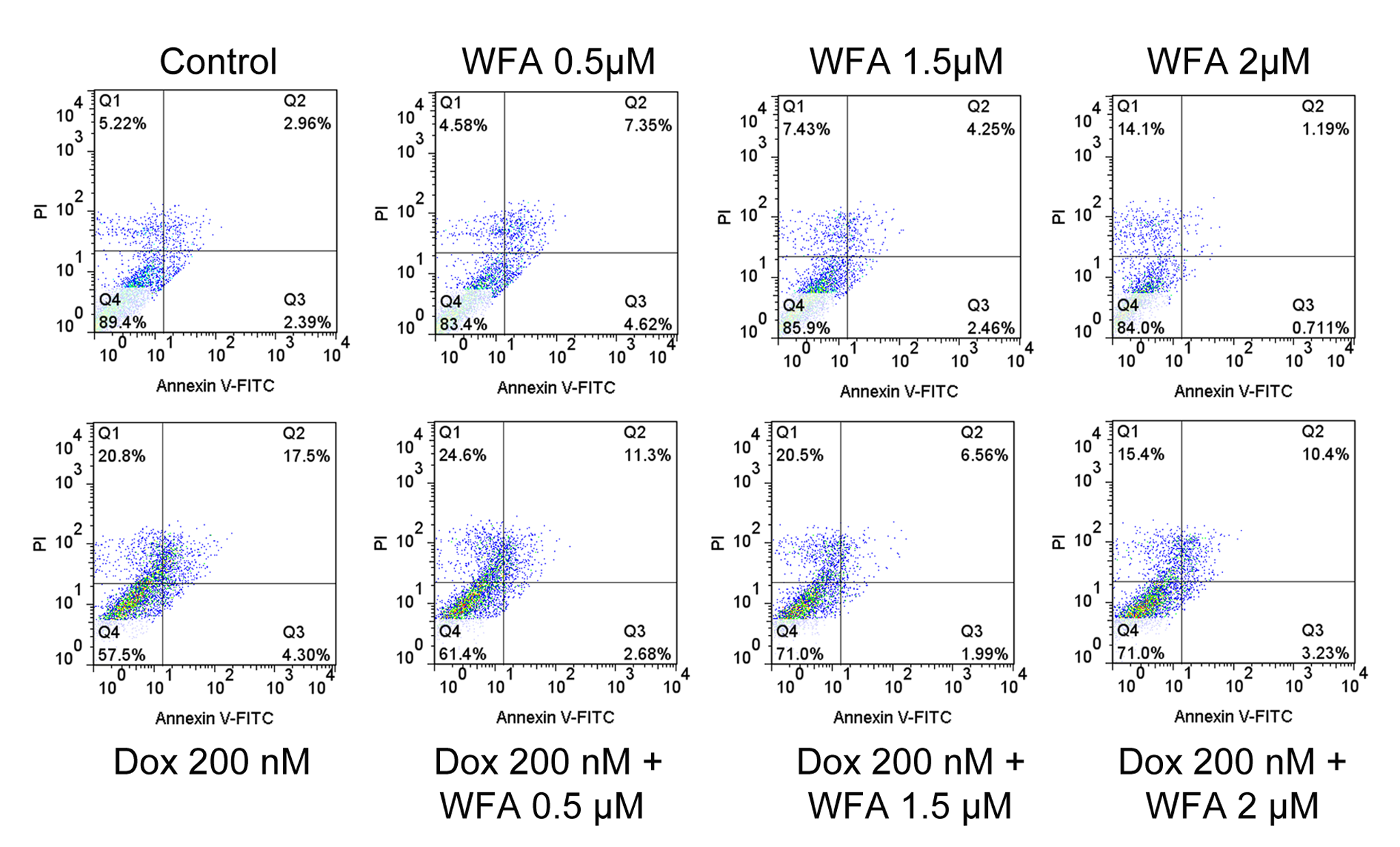

Supplement: Figure S3 — Flow cytometry for Annexin V-FITC for cells treated with Dox and WFA both alone or in combination. A2780 cells were treated with Dox and WFA both alone or combination of WFA/Dox for 24 h as described in Figure 1, dissociated with versene, and stained with Annexin V-FITC and PI. Samples were run on a FACSCaliber and analysis was performed with FlowJo software. (TIF) [file pone.0042265.s003.tif]

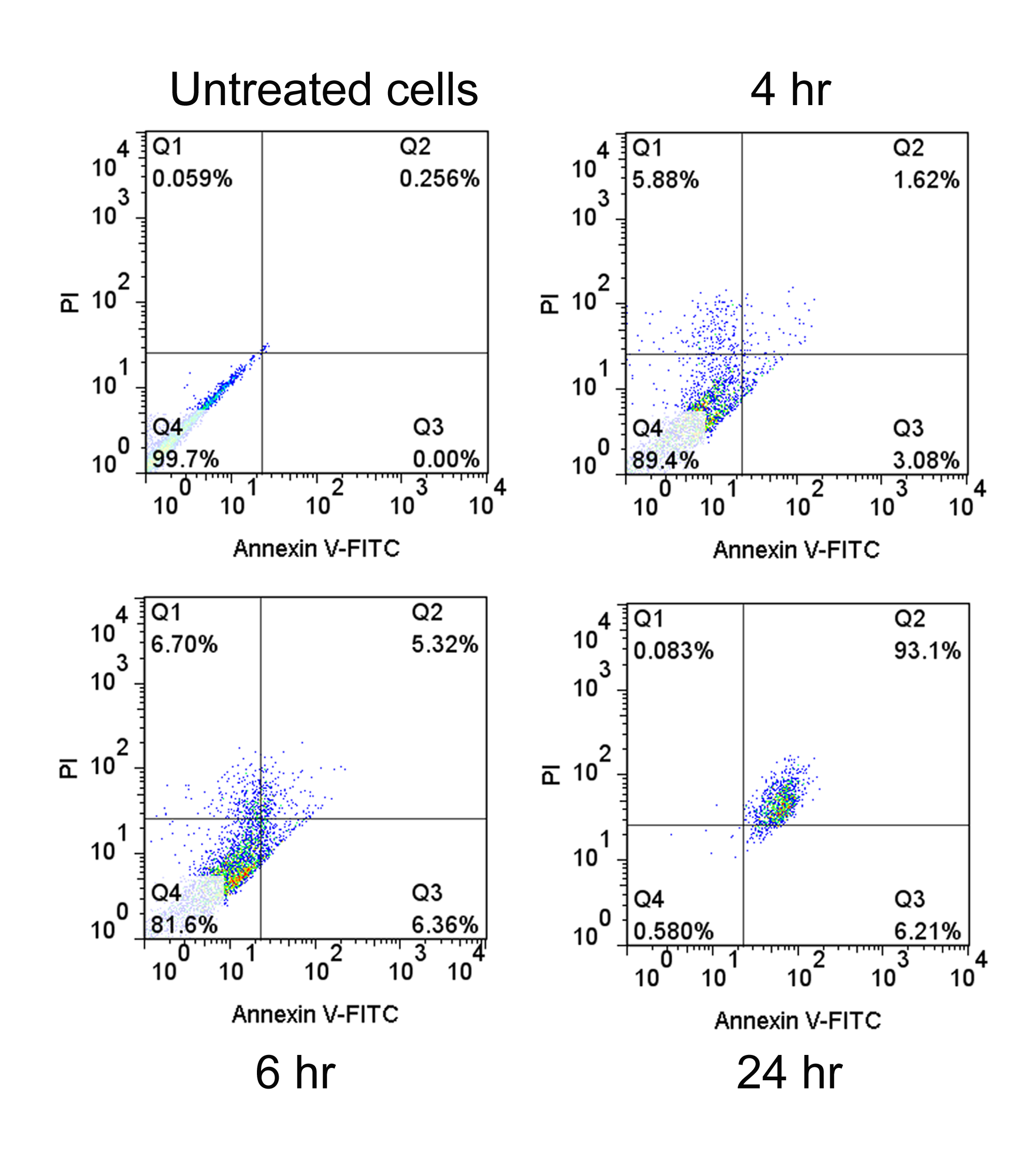

Supplement: Figure S4 — Flow cytometry for Annexin V-FITC for UV treated cells. A2780 cells were exposed to UV for 30 sec and harvested with versene after indicated time and then stained with Annexin V-FITC and PI. Samples were run on a FACSCaliber and analysis performed with FlowJo software. (TIF) [file pone.0042265.s004.tif]

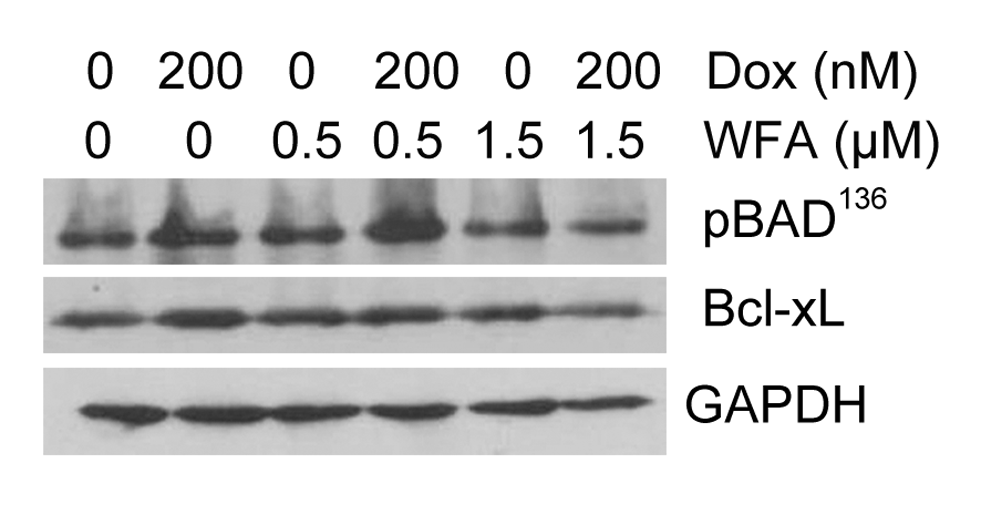

Supplement: Figure S5 — Western blot analysis of intrinsic apoptosis proteins of A2780 cells treated for 24 hr. (TIF) [file pone.0042265.s005.tif]
